# A modified perfusion protocol for pulmonary endarterectomy in a patient with a hematologic malignancy treated with a tyrosine kinase inhibitor

**DOI:** 10.1177/02676591211052161

**Published:** 2021-12-28

**Authors:** Renard G Haumann, Dedré Buys, Eline Hofland, Hans WA Romijn, Suzanne K Kamminga, Jurjan Aman, Esther J Nossent, Petr Symersky

**Affiliations:** 1 Department of Clinical Perfusion, 1209 VUMC Amsterdam University Medical Center, Amsterdam, The Netherlands; 2 Department of Anesthesiology, 1209 VUMC Amsterdam University Medical Center, Amsterdam, The Netherlands; 3 Department of Pulmonology, 1209 VUMC Amsterdam University Medical Center, Amsterdam, The Netherlands; 4 Department of Cardiothoracic Surgery, 10215 OLVG Hospital, Amsterdam, The Netherlands

**Keywords:** pulmonary endarterectomy, chronic myelogenous leukemia, tyrosine, kinase inhibitor, chronic thromboembolic pulmonary hypertension, cardiopulmonary bypass

## Abstract

Tyrosine kinase inhibitors (TKI) are known to be highly effective in the treatment of various cancers with kinase-domain mutations such as chronic myelogenous leukemia. However, they have important side effects such as increased vascular permeability and pulmonary hypertension. In patients undergoing pulmonary endarterectomy with deep hypothermic circulatory arrest, these side effects may exacerbate postoperative complications such as reperfusion edema and persistent pulmonary hypertension. We report on a simple modification of the perfusion strategy to increase intravascular oncotic pressure by retrograde autologous priming and the addition of packed cells and albumin in a patient treated with a TKI.

## Introduction

Chronic thromboembolic pulmonary hypertension (CTEPH) may develop in around 1.5% of patients after acute pulmonary emboli and results from obstruction of vessels by organized clots.^1–5^ Pulmonary endarterectomy (PEA) remains the treatment of choice for CTEPH in patients with surgically accessible disease as it is potentially curative.^3–5^ Patients undergoing PEA represent around 1.7 per million per year of the population in Europe and 0.9 per million in the US. Expert centers report in-hospital mortality rates of < 5% with a mortality rate of 3% and 7% at 3 and 12 months, respectively.^1–6^

PEA is carried out under deep hypothermic circulatory arrest (DHCA) in order to achieve a bloodless surgical field. Stringent perioperative fluid management is paramount in reducing postoperative capillary leak, pulmonary edema, and persistent pulmonary hypertension.^3,4,7^ Tyrosine kinase inhibitors (TKIs) may increase vascular permeability and are associated with pulmonary hypertension and thus may pose a particular risk to patients undergoing PEA.^8–11^ Increased vascular permeability may lead to increased pulmonary reperfusion edema, hypoxia, and persistent pulmonary hypertension postoperatively. We report on a modification of the perfusion protocol to mitigate the effect of a TKI in a patient undergoing PEA.

## Case report

A 45-year-old patient with chronic myelogenous leukemia (CML) was referred to our center for PEA. Massive pulmonary embolism 7 months prior to surgery was treated with rivaroxaban. In addition, she was diagnosed with CML with a blast crisis which prompted treatment with dasatinib, a TKI. The blast crisis subsided with dasatinib. She had persistent dyspnea. One month before referral, a computed tomography angiography (CTA) showed a proximal occlusion of the right pulmonary artery (CTEPH disease level 1C). An allogenic stem cell transplant was considered the only curative treatment option. However, CTEPH precluded a stem cell transplant and the patient was referred for PEA. Mean pulmonary artery pressure was 28 mmHg with a pulmonary vascular resistance of 160 dynes-sec/cm^5^ which indicates mild pulmonary hypertension. The pulmonary wedge pressure was 8 mmHg. Prior to surgery, dasatinib was substituted for nilotinib, a different TKI, which is associated with less capillary leak. RV function was normal, and there were no other comorbidities.

A C5 heart-lung machine with a phophorylcholine-coated tubing-pack, Inspire 8 Oxygenator with integrated arterial filter, and centrifugal pump were used. The following perfusion factors that could have an influence on the volume management in this specific case were identified: colloid osmotic pressures, temperature management during DHCA, and total bypass time.

We removed 400 ml of the prime with retrograde autologous priming (RAP) and added two packed cells with 200 ml of albumin in order to increase intravascular oncotic pressure and aid in the extraction of the fluid from the intracellular and interstitial compartments (Table 1).

**Table 1.** Comparison of the standard San Diego and Amsterdam PEA prime solution to the modified prime for PEA with a TKI. Despite the larger priming volume, a negative fluid balance was achieved.

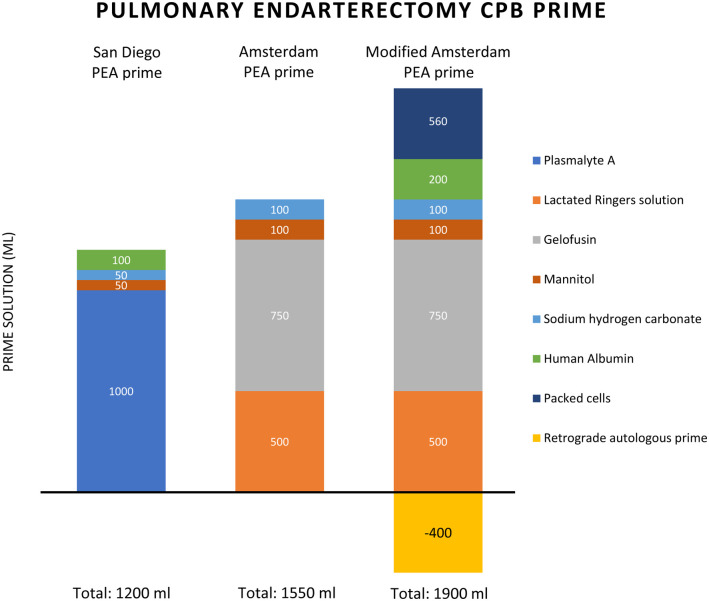

**Figure 1.**
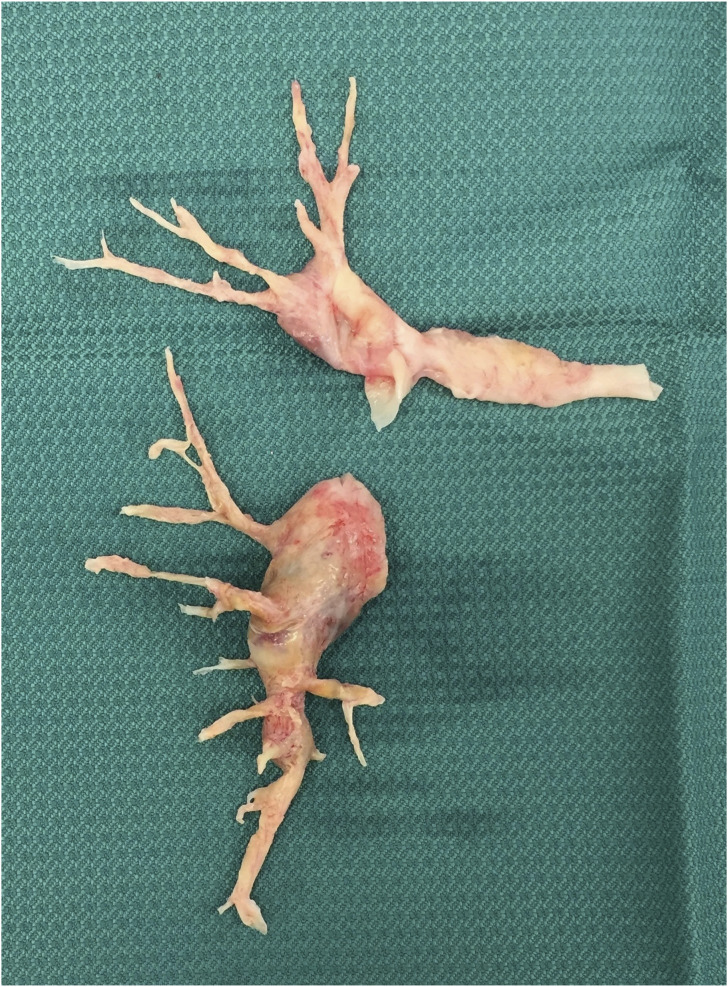
The endarterectomy specimen from the right pulmonary artery.

Cooling was initiated immediately after starting bypass. There were no signs of interstitial fluid loss and cooling was continued to a rectal temperature of 25°C instead of the customary 20°C to reduce bypass time. A cardiac index of 2.4L/min/m2 and a temperature gradient of 10°C were maintained for both the cooling and rewarming phases. Acidbase balance was regulated by α-stat strategy and PaCO_2_ was upregulated to 50 mmHg to facilitate cerebral perfusion during cooling. No additional volume was added during CPB. During DHCA, complete endarterectomy was performed in 12 min (Figure 1) and the CPB was restarted and the patient warmed to normothermia. Hematocrit was 33% pre-CPB, decreased to 25% during CBP and increased to 38% after CPB.

Post-CPB, the patient had a negative fluid balance of −980 ml which was achieved primarily through urine output. We also took into account the prime removed by RAP, external suction, and cellsaver losses. No ultrafiltration was used, and no additional volume was added during CPB.

Total perfusion time was 122 min with a cross clamp time of 41 min. She was extubated within 5 hours. Postoperative course was uneventful, except for drainage of pericardial fluid 5 days postoperatively.

## Discussion

TKIs are a cornerstone in the treatment of hematologic malignancies such as CML. Dasatinib, a second generation TKI, is associated with capillary leak, pulmonary hypertension, and pleural effusion even at low dosages. To our knowledge, there is no report of TKIs in the setting of extra-corporeal circulation. TKIs could be hazardous with the use of extra-corporeal circulation due to the systemic inflammatory response caused by surface activation of the bypass circuitry. Priming and DHCA are additional risk factors that could potentially contribute to capillary leak. These side effects could compound known complications of pulmonary endarterectomy such as pulmonary reperfusion edema, shunting, and persistent pulmonary hypertension.

These potential side effects of TKIs prompted us to modify our standard perfusion protocol for PEA. The strategy consisted of changing the type of TKI, adding colloid solutions, limiting the degree of hypothermia and reducing CPB and operative time. The negative perioperative fluid balance was achieved despite a larger priming volume (Table 1). We observed no signs of capillary leak during the procedure, and the patient could be weaned off the ventilator successfully.

For this patient, several factors may have mitigated the capillary leakage. There was only mild CTEPH and unilateral thromboembolic disease which allowed modification of temperature and shorter arrest times. In addition, it was possible to switch the TKI from dasatinib to nilotinib which is associated with less capillary leak.

## Conclusion

PEA may be feasible with an adapted perfusion protocol in patients treated with TKIs. Increased intravascular oncotic pressure was achieved by retrograde autologous priming and the addition of packed cells and albumin.
